# Long-term safety and efficacy of ropeginterferon alfa-2b in Japanese patients with polycythemia vera

**DOI:** 10.1007/s12185-024-03846-5

**Published:** 2024-10-03

**Authors:** Keita Kirito, Yuka Sugimoto, Akihiko Gotoh, Katsuto Takenaka, Michiko Ichii, Tadaaki Inano, Shuichi Shirane, Masafumi Ito, Oleh Zagrijtschuk, Albert Qin, Hiroaki Kawase, Toshiaki Sato, Norio Komatsu, Kazuya Shimoda

**Affiliations:** 1 Department of Hematology and Oncology, University of Yamanashi, 1110 Shimokato, Chuo, Yamanashi 409-3898 Japan; 2 Department of Hematology and Oncology, Mie University Graduate School of Medicine, 2-174 Edobashi, Tsu, Mie 514-8507 Japan; 3 Department of Hematology, Tokyo Medical University, 6-7-1 Nishishinjuku, Shinjuku-ku, Tokyo, 160-0023 Japan; 4 Department of Hematology, Clinical Immunology and Infectious Diseases, Ehime University Graduate School of Medicine, 454 Shitsukawa, Toon, Ehime 791-0295 Japan; 5 Department of Hematology and Oncology, Osaka University Graduate School of Medicine, 2-2 Yamadaoka, Suita, Osaka 565-0871 Japan; 6 Department of Hematology, Juntendo University Graduate School of Medicine, 2-1-1 Hongo, Bunkyo-ku, Tokyo, 113-8421 Japan; 7 Department of Advanced Hematology, Juntendo University Graduate School of Medicine, 2-1-1 Hongo, Bunkyo-ku, Tokyo, 113-8421 Japan; 8 Department of Pathology, Japanese Red Cross Aichi Medical Center Nagoya Daiichi Hospital, 3-35 Michishita-cho, Nakamura-ku, Nagoya, Aichi 453-8511 Japan; 9 PharmaEssentia Corporation USA, 35 Corporate Drive, Suite 325, Burlington, MA 01803 USA; 10 Medical Research and Clinical Operations, PharmaEssentia Corporation, 13F, No. 3, YuanQu Street, Nangang District, Taipei, 115 Taiwan; 11 PharmaEssentia Japan KK, Akasaka Center Building 12F, 1-3-13 Moto-akasaka, Minato-ku, Tokyo, 107-0051 Japan; 12 Division of Hematology, Diabetes and Endocrinology, Department of Internal Medicine, Faculty of Medicine, University of Miyazaki, 5200 Kiyotakecho Kihara, Miyazaki, Miyazaki 889-1692 Japan

**Keywords:** Hematologic response, *JAK2* V617F allele burden, Molecular response, Polycythemia vera, Ropeginterferon alfa-2b

## Abstract

Ropeginterferon alfa-2b (ropegIFN), a new-generation interferon-based agent, has been approved in Japan for patients with polycythemia vera (PV) who are ineligible for or respond inadequately to conventional treatment. However, long-term outcomes with ropegIFN in Japanese patients have not been reported. This extension of a phase 2 study of ropegIFN in Japanese patients with PV aimed to determine its long-term safety/efficacy, and changes over time in *JAK2* V617F allele burden. Here, we report data from the phase 2 study and subsequent extension over a period of 36 months. The primary endpoint was the complete hematologic response (CHR) maintenance rate without phlebotomy (hematocrit value < 45% without phlebotomy during the previous 12 weeks, platelet count ≤ 400 × 10^9^/L, and white blood cell count ≤ 10 × 10^9^/L). The CHR maintenance rates were 8/27 (29.6%), 18/27 (66.7%), and 22/27 (81.5%) at 12, 24, and 36 months, respectively. No thrombotic or hemorrhagic events occurred. The median allele burden change from baseline was − 74.8% at 36 months. All patients experienced adverse events; 25/27 (92.6%) experienced adverse drug reactions (ADRs), but no serious ADRs or deaths occurred. This interim analysis demonstrated the safety and efficacy of ropegIFN over 36 months in Japanese patients with PV.

**Supplementary Information:**

The online version contains supplementary material available at 10.1007/s12185-024-03846-5.

## Introduction

Polycythemia vera (PV), one of the Philadelphia chromosome-negative myeloproliferative neoplasms (MPNs), is characterized by overproduction of red blood cells (RBCs), which can lead to thrombotic and hemorrhagic events [1, 2]. Patients with PV also have an increased risk of developing acute myeloid leukemia or myelofibrosis [1, 2], and the development of these conditions contributes to worsening prognosis in affected patients [3, 4]. The majority of PV cases have an acquired gain-of-function mutation in the Janus kinase 2 (*JAK2*) gene (*JAK2* V617F) [1]. A high *JAK2* V617F allele burden is associated with thrombotic events and transition to myelofibrosis [2, 5]. Although most patients with PV are diagnosed later in life (median age of diagnosis: 61 years; median age of diagnosis in Japan: 65 years), PV can also manifest in younger patients [2, 6].

The effectiveness of interferon (IFN) for the treatment of PV was first reported in the 1980s [7] and pegylated IFN has been available since the 2000s. Current management of PV includes phlebotomy and low-dose aspirin as first-line treatment, with the addition of cytoreductive therapy (hydroxyurea [HU] or pegylated IFN) recommended for patients at a higher risk of thrombosis, and ruxolitinib (a JAK1 and JAK2 inhibitor) as second-line treatment [8–11]. However, treatment options in Japan have been limited, as earlier IFN therapies, including peginterferon alfa-2a, were not included in Japanese insurance coverage for the treatment of MPN.

Ropeginterferon alfa-2b (ropegIFN) is a novel, site-selective, monopegylated recombinant human IFN [12]. A phase 1/2 study (PEGINVERA) [12], a phase 3 study (PROUD-PV) and its extension study (CONTINUATION-PV) [13, 14] have determined the maximum tolerated dose and reported that ropegIFN is both effective and well tolerated in patients with PV. Based on the results of these clinical studies, ropegIFN has been recommended for patients with high risk of thrombosis and for patients who require cytoreductive therapy, even with a low risk of thrombosis, in some guidelines [8, 10]. In Japan, a phase 2 study of ropegIFN was reported [15], and it was recently approved for patients with PV who have an inadequate response to conventional treatment or for whom conventional treatment is inappropriate [16], including younger patients who are expected to require long-term treatment.

Long-term control of hematocrit levels, platelet counts, and white blood cell (WBC) counts is necessary to lower the risk of thrombosis and bleeding in patients with PV [1, 2, 17]. Although long-term data in non-Japanese populations have been reported [13, 14], Japanese data are limited to those obtained from a 12-month phase 2 study [15]. Because of the necessity for long-term safety and efficacy data in the Japanese population, an extension study of the phase 2 study was conducted. This article reports an interim analysis of the extension study, and includes a total of 36 months of data (collected during the original 12-month phase 2 study, plus 24 months of data from the extension study) with the aim of elucidating the safety and efficacy of long-term treatment with ropegIFN and the change over time in *JAK2* V617F allele burden.

## Materials and methods

### Study design and treatments

This extension study of a phase 2, open-label, multicenter, single-arm study investigated the safety and efficacy of long-term ropegIFN treatment in Japanese patients with PV; the design and results of the phase 2 study have been published previously [15, 18]. The initial study (NCT04182100) was conducted between December 2019 and April 2021. In this report, we analyzed data up to 36 months of treatment.

The study was conducted in compliance with the ethical principles that have their origins in the Declaration of Helsinki, the International Council for Harmonisation Good Clinical Practice regulations, and other relevant regulatory requirements. The study protocol was approved by the institutional review boards at each study site, and all patients provided written informed consent prior to participation. Additional written informed consent was obtained prior to participation in the extension study.

The treatment regimen has been previously described [15, 18]. Patients maintained the dose of ropegIFN that they received at the end of the phase 2 study [15], in which the overall mean ± standard deviation (SD) dose administered once every 2 weeks was 396.3 ± 133.0 μg; 14/29 patients (48.3%) reached the maximum dose (500 µg), with more than half of these patients achieving this dose by week 20. The interval between doses was 2 weeks; dose intervals of 3 or 4 weeks were allowed for patients who achieved a complete hematologic response (CHR) without requiring phlebotomy by month 12 (defined as a durable response at weeks 36 and 52) in the original study, and those who achieved CHR without requiring phlebotomy during the extension study. Patients also received concomitant low-dose (75–150 mg/day) aspirin, unless contraindicated. Patients underwent phlebotomy if their hematocrit reached ≥ 45%. The study treatment is intended to continue until disease progression, death, or patient withdrawal.

### Patients

The eligibility/exclusion criteria have been published previously [15, 18]. Patients aged ≥ 20 years with a diagnosis of PV who had completed the 12-month treatment period in the phase 2 study were eligible for participation in the extension study. Patients were excluded from continuing to the extension study if the investigator or subinvestigator determined that continued administration of ropegIFN was inappropriate.

### Study assessments

Assessments were performed at both the local laboratory and a central laboratory. Local laboratory assessments were planned every 6 or 8 weeks and central efficacy measurements every 12 weeks. The primary endpoint was the CHR maintenance rate without phlebotomy, defined as hematocrit values of < 45% without phlebotomy during the previous 12 weeks, platelet count ≤ 400 × 10^9^/L, and WBC count ≤ 10 × 10^9^/L. This rate was evaluated at 52-week intervals.

Secondary endpoints included change over time in hematocrit values; WBC, RBC, and platelet counts every 52 weeks; change over time in spleen size (by ultrasound imaging); presence or absence of phlebotomy; proportion of patients without thrombotic or hemorrhagic events; and change over time in *JAK2* V617F allele burden. Assessment of *JAK2* V617F allele burden was conducted using an ipsogen^®^ JAK2 MutaQuant^®^ kit (QIAGEN^®^, Hilden, Germany) [19, 20] on genomic DNA from venous blood samples collected prior to ropegIFN administration. Patients were informed of the genetic test results if they requested disclosure.

### Safety assessments

Safety outcomes included adverse events (AEs), serious AEs (SAEs), treatment-emergent AEs (TEAEs), and adverse drug reactions (ADRs), which were categorized using the Medical Dictionary for Regulatory Activities version 26.0 and assessed according to the Common Terminology Criteria for Adverse Events version 5.0. AEs of special interest (AESIs), including immune reactions and cardiovascular, hemorrhagic, thrombotic, psychiatric, and ocular AEs, were also recorded. All AEs reported on the date of the first dose until 30 days after the last dose were considered TEAEs. Patients with multiple AEs were counted once for the most severe occurrence of each type of event.

### Statistical methods

All patients who completed 12 months of treatment during the phase 2 study [15, 18] and agreed to continue treatment in the extension study were included. No blinding or randomization was performed as this was a single-arm study, and the intention-to-treat (ITT) population and the safety population comprised all patients who received at least one dose of ropegIFN.

Continuous variables were summarized by number, mean, median, SD, interquartile range, and minimum and maximum values, and categorical variables were summarized by number, frequency, and percentage. The denominator for percentages was the total number of patients. Using the CHR rate at month 12 (defined as a durable response at weeks 36 and 52), CHR maintenance rates for each subsequent 12-month period and their 95% confidence intervals were calculated. Patients were stratified by age (< 60/ ≥ 60 years), use of prior HU treatment (yes/no), and the median disease duration, defined as the diagnosis date to the date of the first dose (< 3.1/ ≥ 3.1 years). In the PROUD-PV and CONTINUATION-PV studies, early PV was determined as patients who had received no previous cytoreductive treatment or fewer than 3 years of previous HU treatment [13]; thus, the disease duration cut-off value in this study distinguished patients with early PV. Incomplete dates and times were imputed for the duration of AEs; other missing data and outliers were not imputed.

All statistical analyses were conducted using SAS version 9.4 or higher (SAS Institute Inc., Cary, NC, USA).

## Results

### Patients

All 27 patients who completed the phase 2 study were included in this extension study, and all patients were evaluated in this interim analysis. The baseline characteristics are summarized in Table S1. Of the evaluated patients, 16 (59.3%) were female and 11 (40.7%) were male, and the median (range) age was 54 (26–72) years. All patients had an Eastern Cooperative Oncology Group grade of 0, 13 (48.1%) had prior HU use, and 13 (48.1%) had a disease duration < 3.1 years.

### Maintenance of complete hematologic response

**Fig. 1:**
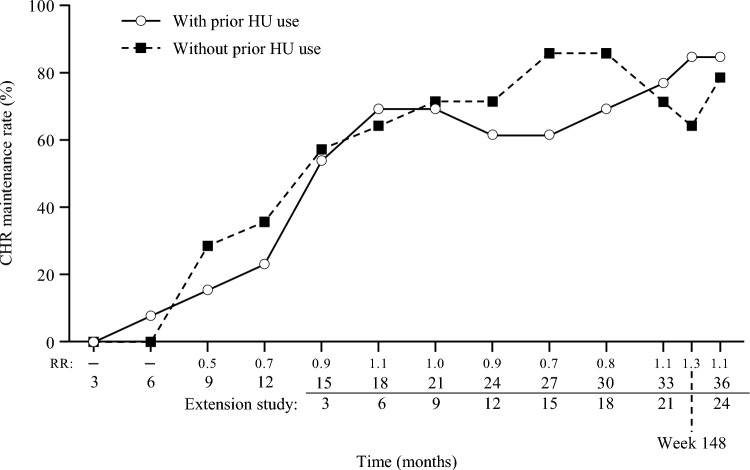
Maintenance of CHR with/without prior HU use (intention-to-treat population, central laboratory data). *CHR*, complete hematologic response; *HU*, hydroxyurea; *RR*, relative risk

The maintenance rates of CHR were 8/27 (29.6%), 18/27 (66.7%), and 22/27 (81.5%) at 12 months, 24 months (extension month 12), and 36 months (extension month 24), respectively. The observed increase over time in the maintenance rate of CHR occurred regardless of the presence/absence of prior HU treatment (Fig. 1). Assessments from the central and local laboratories showed similar results (Fig. S1).

**Fig. 2:**
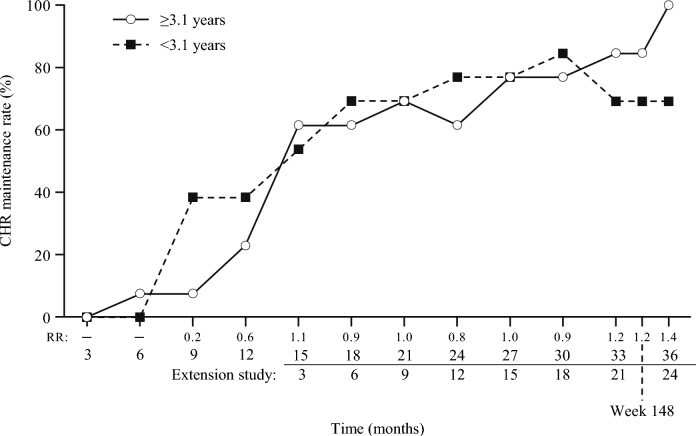
Maintenance of CHR by disease duration (intention-to-treat population, central laboratory data). *CHR*, complete hematologic response; *RR*, relative risk

When patients were stratified by disease duration (< 3.1/ ≥ 3.1 years), there was a similar trend regarding the maintenance rate of CHR over time (Fig. 2). Approximately 70% of patients with a disease duration of < 3.1 years, and 100% of those with a disease duration ≥ 3.1 years, maintained CHR status at 36 months. Similar maintenance rates of CHR over time were also observed in patients < 60 and those ≥ 60 years of age (Fig. S2).

### Secondary outcomes

The median (percentage) changes from baseline in hematocrit values were − 3.5% (− 7.4%), − 7.6% (− 15.2%), and − 9.8% (− 20.5%) at 12 months, 24 months, and 36 months, respectively. Regarding WBC counts, the median (percentage) changes from baseline were − 10.3 × 10^9^/L (− 63.5%), − 10.5 × 10^9^/L (− 68.9%), and − 10.0 × 10^9^/L (− 67.4%) at 12 months, 24 months, and 36 months, respectively. The median (percentage) changes from baseline in platelet count were − 383.0 × 10^9^/L (− 65.2%), − 410.0 × 10^9^/L (− 68.8%), and − 397.0 × 10^9^/L (− 70.5%) at 12 months, 24 months, and 36 months, respectively. Changes in these parameters are shown graphically in Fig. S3. No thrombotic or hemorrhagic events were reported.

Changes from baseline in spleen size are described in Table S2. The median (percentage) changes from baseline were − 2.1 cm^2^ (− 4.2%), − 6.1 cm^2^ (− 13.1%), and − 9.2 cm^2^ (− 15.5%) at 12 months, 24 months, and 36 months, respectively.

Throughout the study period, 12 patients received ropegIFN every 2 weeks, and 15 patients received ropegIFN in 3- or 4-week intervals. Fourteen patients received the maximum dose (500 µg) (Table S3 and Fig. S4). Five patients self-administered ropegIFN at least once during the study period.

**Fig. 3:**
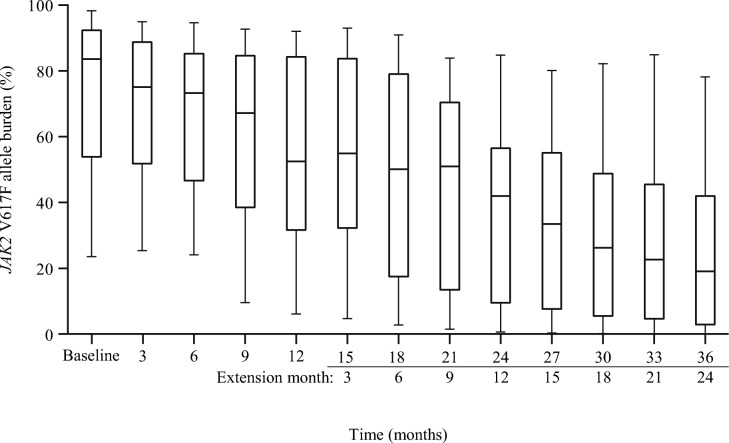
*JAK2* V617F allele burden (intention-to-treat population). The box shows the median line and upper and lower quartiles, whiskers represent the range. Data at week 148 or week 160 are shown as 36 months, as one patient had missing data at week 148; therefore, week 160 data were used for this patient

The *JAK2* V617F allele burden over time is shown in Fig. 3. The median *JAK2* V617F allele burden change from baseline was − 74.8% at the last observation point (36 months), and the median (range) allele burden was 83.6% (23.6–98.3%) at baseline and 19.3% (0.1–79.7%) at the last observation point.

Among the 27 patients enrolled in the extension study, one patient was negative for *JAK2* V617F at baseline. Changes in allele burden throughout the study for each of the 26 patients with a *JAK2* V617F mutation at baseline are illustrated in Fig. S5. At month 36, 34.6% (9/26) of patients had a *JAK2* V617F allele burden ≤ 10% (Fig. S5A), all of whom achieved a CHR. In two of these patients, the allele burden declined below the detectable limit at 36 months. In the other 17 patients, the *JAK2* V617F allele burden tended to decline over time, but remained > 10% at 36 months (Fig. S5B). Of the 17 patients with a *JAK2* V617F burden > 10% at 36 months, 14 achieved a CHR, two had hematocrit < 45% (without prior phlebotomy within the previous 12 weeks), and one had a platelet count ≤ 400 × 10^9^/L.

### Safety

A summary of TEAEs and ADRs, including those previously reported at 12 months and those with an onset during the extension study, is shown in Table 1. All patients (27/27 [100.0%]) experienced at least one TEAE and 7/27 (25.9%) experienced grade 3 or higher TEAEs (Table S4). Most patients (25/27 [92.6%]) experienced at least one ADR; 8/27 (29.6%) patients experienced a grade 2 ADR and 2/27 (7.4%) experienced a grade 3 ADR. No transitions to acute myeloid leukemia or myelofibrosis occurred, and no grade 4 or 5 TEAEs or ADRs were reported. Three patients experienced a combined total of five SAEs. The most common ADRs included decreased WBC count (7/27 [25.9%]) and increased urinary beta-2 microglobulin (7/27 [25.9%]). Alopecia, the most common ADR at 12 months (16/29 [55.2%]), was reported in only one patient in the current analysis (1/27 [3.7%]).

**Table 1:** Summary of TEAEs and ADRs at 12 months [15]^a^ vs those occurring from > 12–36 months^b^ (safety population)

	Phase 2 study [15] (up to 12 months) (*N* = 29)	Extension study (> 12–36 months) (*N* = 27)
At least one ADR	29 (100)	25 (92.6)
At least one grade 1 ADR	20 (69.0)	15 (55.6)
At least one grade 2 ADR	9 (31.0)	8 (29.6)
At least one grade ≥ 3 ADR	0	2 (7.4)
At least one serious TEAE	1 (3.4)	3 (11.1)
At least one TEAE of special interest	9 (31.0)	7 (25.9)
ADR leading to treatment discontinuation	1 (3.4)	0
*ADRs occurring in* ≥ *10% of patients (in either study) by Preferred Term*		
Alopecia	16 (55.2)	1 (3.7)
Fatigue	8 (27.6)	1 (3.7)
Influenza-like illness	8 (27.6)	0
Alanine aminotransferase increased	6 (20.7)	1 (3.7)
Beta-2 microglobulin urine increased	6 (20.7)	7 (25.9)
Aspartate aminotransferase increased	5 (17.2)	1 (3.7)
Diarrhea	5 (17.2)	1 (3.7)
Liver function test abnormal	4 (13.8)	1 (3.7)
Myalgia	4 (13.8)	3 (11.1)
Pyrexia	4 (13.8)	2 (7.4)
Anemia	3 (10.3)	5 (18.5)
Arthralgia	3 (10.3)	0
Gamma glutamyl transferase increased	3 (10.3)	0
White blood cell count decreased	0	7 (25.9)
Injection site reaction	3 (10.3)	0
Malaise	3 (10.3)	3 (11.1)
*TEAEs of special interest by System Organ Class and Preferred Term*		
Endocrine disorders	3 (10.3)	2 (7.4)
Hypothyroidism	2 (6.9)	2 (7.4)
Silent thyroiditis	1 (3.4)	0
Psychiatric disorders	2 (6.9)	0
Anxiety	1 (3.4)	0
Suicidal ideation	1 (3.4)	0
Eye disorders	1 (3.4)	1 (3.7)
Retinal hemorrhage	1 (3.4)	1 (3.7)

Data are *n* (%)

Number and percentage of TEAEs in the present study represent new events confirmed during the extension study (i.e., from > 12–36 months); events that occurred during the original 12-month study were not included. Patients who experienced an event during the original study that resolved and then experienced another event during the extension study were included.

*ADR*, adverse drug reaction; *TEAE*, treatment-emergent adverse event

^a^Medical Dictionary for Regulatory Activities, v23.0

^b^Medical Dictionary for Regulatory Activities, v26.0

Regarding HU treatment, 5/13 (38.5%) patients with prior HU treatment and 2/14 (14.3%) without prior HU treatment experienced grade 3 or higher TEAEs (Table S4). Among patients with prior HU treatment, one (7.7%) had an AESI, which was judged to be unrelated to the study drug. Among those without prior HU treatment, 6/14 (42.9%) patients had an AESI and 4 (28.6%) of these were considered related to the study drug. AESIs included hypothyroidism (2/27 [7.4%]) and arteriosclerotic retinopathy, retinal hemorrhage, hypergammaglobulinemia, enteritis infectious, and adjustment disorder (all 1/27 [3.7%]).

One patient discontinued treatment because of prostate cancer, which was considered unrelated to the study drug. A summary of the details of dose adjustments and interruptions, including AEs considered related to dose reductions and interruptions, is provided in Table S5. No ADRs led to discontinuations, and no serious ADRs or deaths were reported. ADRs leading to dose reduction occurred in 10/27 patients (37.0%), including a 50-μg dose reduction (i.e., dose reduction to the dose of 50 μg) in three patients (11.1%), a 100-μg dose reduction in two patients (7.4%), and ≥ 150-μg dose reductions in five patients (18.5%). ADRs leading to dose interruption occurred in 5/27 patients (18.5%), including one interruption in two patients (7.4%), two interruptions in two patients (7.4%), and three or more interruptions in one patient (3.7%).

## Discussion

In the main phase 2 study, 8/29 (27.6%) patients achieved a CHR. This extension study included 27 patients who completed the phase 2 study [15]; two patients from the phase 2 study were not included in the extension (one patient discontinued treatment due to silent thyroiditis and one patient withdrew consent). The data from this analysis showed that those patients who achieved a CHR during the phase 2 study maintained their CHR status over 36 months. Furthermore, 14 patients who had not achieved a CHR in the phase 2 study achieved a CHR following long-term administration in the extension study, suggesting that continuous long-term treatment with ropegIFN improves efficacy outcomes. These results were consistent regardless of the presence or absence of prior HU treatment, the duration of disease, or the age of the patient. Thus, a therapeutic effect was observed regardless of patient characteristics and was maintained long-term. Moreover, there was a favorable reduction in *JAK2* V617F allele burden over time, with an allele burden change from baseline of − 74.8% at the last observation point. The long-term maintenance of CHR status combined with the reduction in allele burden show that prolonged ropegIFN treatment can lead to a durable response and greater efficacy than short-term treatment, and may lead to an operational cure for PV (defined as a *JAK2* V617F allele burden ≤ 10%, a CHR maintained for ≥ 2 years, and no disease progression, thromboembolic events, or worsening of symptoms) [8]. Regarding safety, no new or unexpected ADRs were reported. These results provide evidence for the long-term tolerability and safety of ropegIFN. Overall, this analysis demonstrated the efficacy and safety of ropegIFN over 36 months in Japanese patients with PV, including those at low risk of thrombosis who do not respond to currently recommended treatments [11].

No differences in CHR achievement rates were observed when patients were stratified by age, prior HU treatment, or disease duration. However, it should be noted that while half of the study population had a disease duration longer than 3.1 years at baseline and half had a history of HU treatment, only six patients (22.2%) in this study were aged ≥ 60 years.

The maintenance rate of CHR in this study was 29.6% at 12 months; 26/27 patients completed 36 months of ropegIFN treatment and reached a maintenance rate of CHR of 81.5%. In the CONTINUATION-PV study [13], 53/123 (43.1%) patients with PV who received ropegIFN achieved a CHR at 12 months; this increased to 67/95 (70.5%) after 36 months of treatment, which is consistent with our findings. Moreover, patients in the current study had both a longer median duration of disease (this study: 3.1 years, CONTINUATION-PV: 1.8 months) and a higher *JAK2* V617F allele burden at baseline (this study: median 83.6%, CONTINUATION-PV: mean 42.8%) than patients in the CONTINUATION-PV study [13]. In addition, no thrombotic or hemorrhagic events were reported in this analysis, and no patients developed acute myeloid leukemia or myelofibrosis. Overall, these studies have confirmed the efficacy and tolerability of ropegIFN, regardless of patient background factors.

As presence of the *JAK2* V617F allele is a risk factor for poor outcomes, including thrombosis [5, 6, 21], treatments that reduce the *JAK2* V617F allele burden are anticipated to improve outcomes for patients with PV. Ruxolitinib (as assessed in the MAJIC-PV study) has been reported to reduce *JAK2* V617F allele burden; furthermore, reduced allele burden was associated with improved progression-free and event-free survival in patients who received either ruxolitinib or the best available therapy [22]. As ropegIFN reduces the *JAK2* V617F allele burden, it is likely that ropegIFN may prevent the progression of PV. This is supported by the findings from the CONTINUATION-PV study, which suggested that long-term treatment with ropegIFN resulted in both CHR and a reduction in allele burden below 1% [23]. Moreover, the disease progression rates with ropegIFN treatment were low, including only one patient who developed myelofibrosis [23]. We observed an increased reduction of the median (range) *JAK2* V617F allele burden at the last observation point (36 months) compared with the results at month 12 (19.3% [0.1–79.7%] vs 52.5% [6.2–92.0%], respectively) [15]. These findings suggest that continued use of ropegIFN may contribute to a reduction in the *JAK2* V617F allele burden and prevent disease progression in patients with PV. Furthermore, the observed reduction in allele burden combined with the increased CHR rate over time support the previous suggestion that long-term ropegIFN treatment may lead to an operational cure for patients with PV [8, 23].

In the phase 2 study, one patient discontinued ropegIFN treatment due to the occurrence of silent thyroiditis [15]. In addition, there were four instances (in three patients) of hypothyroidism that were managed without requiring dose reductions. In this analysis, no new TEAEs led to treatment discontinuations and no new safety concerns were identified over the 36 months. These results suggest that ropegIFN is both an effective and well tolerated option for long-term treatment of PV.

We acknowledge the limitations of this study. While no differences were observed between patients aged < 60 and ≥ 60 years, the small number of patients in the group aged ≥ 60 years limits the strength of inferences that can be made. Furthermore, the study population may not fully reflect the characteristics of patients who may benefit the most from ropegIFN. Nevertheless, the benefits of long-term ropegIFN treatment were observed in a relatively young population, in which patients are likely to require treatment over a longer period of time.

This interim analysis confirmed the efficacy and safety of ropegIFN over 36 months in Japanese patients with PV. No thrombotic or hemorrhagic events or transition to acute myeloid leukemia or myelofibrosis occurred during this study. These findings also indicate that patients with PV who do not achieve CHR within the first 12 months of treatment with ropegIFN may yet obtain clinical benefit from continued administration. As treatment beyond 36 months is likely to occur in real-world clinical practice, observation of the long-term efficacy and safety of ropegIFN will be necessary in the future.

## Supplementary Information

Below is the link to the electronic supplementary material.

Supplementary file1 (PDF 118 kb)

Supplementary file2 (PDF 227 kb)

## Data Availability

The data that support the findings of this study are available on reasonable request from the corresponding author (Keita Kirito) or the sponsor, PharmaEssentia Japan KK.
