# Correction: Long-term safety and efficacy of ropeginterferon alfa-2b in Japanese patients with polycythemia vera

**DOI:** 10.1007/s12185-025-04002-3

**Published:** 2025-05-16

**Authors:** Keita Kirito, Yuka Sugimoto, Akihiko Gotoh, Katsuto Takenaka, Michiko Ichii, Tadaaki Inano, Shuichi Shirane, Masafumi Ito, Oleh Zagrijtschuk, Albert Qin, Hiroaki Kawase, Toshiaki Sato, Norio Komatsu, Kazuya Shimoda

**Affiliations:** 1 Department of Hematology and Oncology, University of Yamanashi, 1110 Shimokato, Chuo, Yamanashi 409-3898 Japan; 2 Department of Hematology and Oncology, Mie University Graduate School of Medicine, 2-174 Edobashi, Tsu, Mie 514-8507 Japan; 3 Department of Hematology, Tokyo Medical University, 6-7-1 Nishishinjuku, Shinjuku-ku, Tokyo, 160-0023 Japan; 4 Department of Hematology, Clinical Immunology and Infectious Diseases, Ehime University Graduate School of Medicine, 454 Shitsukawa, Toon, Ehime 791-0295 Japan; 5 Department of Hematology and Oncology, Osaka University Graduate School of Medicine, 2-2 Yamadaoka, Suita, Osaka 565-0871 Japan; 6 Department of Hematology, Juntendo University Graduate School of Medicine, 2-1-1 Hongo, Bunkyo-ku, Tokyo, 113-8421 Japan; 7 Department of Advanced Hematology, Juntendo University Graduate School of Medicine, 2-1-1 Hongo, Bunkyo-ku, Tokyo, 113-8421 Japan; 8 Department of Pathology, Japanese Red Cross Aichi Medical Center Nagoya Daiichi Hospital, 3-35 Michishita-cho, Nakamura-ku, Nagoya, Aichi 453-8511 Japan; 9 PharmaEssentia Corporation USA, 35 Corporate Drive, Suite 325, Burlington, MA 01803 USA; 10 Medical Research and Clinical Operations, PharmaEssentia Corporation, 13F, No. 3, YuanQu Street, Nangang District, Taipei, 115 Taiwan; 11 PharmaEssentia Japan KK, Akasaka Center Building 12F, 1-3-13 Moto-akasaka, Minato-ku, Tokyo, 107-0051 Japan; 12 Division of Hematology, Diabetes and Endocrinology, Department of Internal Medicine, Faculty of Medicine, University of Miyazaki, 5200 Kiyotakecho Kihara, Miyazaki, Miyazaki 889-1692 Japan

**Correction: International Journal of Hematology (2024) 120:675–683** 10.1007/s12185-024-03846-5

In Table 1 of this article, the data in the “Psychiatric disorders” headed “Phase 2 study [15] …” were incorrect and should have been “1 (3.4)”. The entire row ‘Suicidal ideation’ should be deleted under ‘Psychiatric disorders’.

Uncorrected version of Table 1

**Table 1:** Summary of TEAEs and ADRs at 12 months [15]^a^ vs those occurring from >12–36 months^b^ (safety population)

	Phase 2 study [15] (up to 12 months) (*N *= 29)	Extension study (> 12–36 months) (*N* = 27)
At least one ADR	29 (100)	25 (92.6)
At least one grade 1 ADR	20 (69.0)	15 (55.6)
At least one grade 2 ADR	9 (31.0)	8 (29.6)
At least one grade ≥ 3 ADR	0	2 (7.4)
At least one serious TEAE	1 (3.4)	3 (11.1)
At least one TEAE of special interest	9 (31.0)	7 (25.9)
ADR leading to treatment discontinuation	1 (3.4)	0
*ADRs occurring in ≥* *10% of patients (in either study) by Preferred Term*		
Alopecia	16 (55.2)	1 (3.7)
Fatigue	8 (27.6)	1 (3.7)
Influenza-like illness	8 (27.6)	0
Alanine aminotransferase increased	6 (20.7)	1 (3.7)
Beta-2 microglobulin urine increased	6 (20.7)	7 (25.9)
Aspartate aminotransferase increased	5 (17.2)	1 (3.7)
Diarrhea	5 (17.2)	1 (3.7)
Liver function test abnormal	4 (13.8)	1 (3.7)
Myalgia	4 (13.8)	3 (11.1)
Pyrexia	4 (13.8)	2 (7.4)
Anemia	3 (10.3)	5 (18.5)
Arthralgia	3 (10.3)	0
Gamma glutamyl transferase increased	3 (10.3)	0
White blood cell count decreased	0	7 (25.9)
Injection site reaction	3 (10.3)	0
Malaise	3 (10.3)	3 (11.1)
*TEAEs of special interest by System Organ Class and Preferred Term*		
Endocrine disorders	3 (10.3)	2 (7.4)
Hypothyroidism	2 (6.9)	2 (7.4)
Silent thyroiditis	1 (3.4)	0
Psychiatric disorders	2 (6.9)	0
Anxiety	1 (3.4)	0
Suicidal ideation	1 (3.4)	0
Eye disorders	1 (3.4)	1 (3.7)
Retinal hemorrhage	1 (3.4)	1 (3.7)

Data are *n* (%)

Number and percentage of TEAEs in the present study represent new events confirmed during the extension study (i.e., from > 12–36 months); events that occurred during the original 12-month study were not included. Patients who experienced an event during the original study that resolved and then experienced another event during the extension study were included.

*ADR* adverse drug reaction, *TEAE* treatment-emergent adverse event

^a^Medical Dictionary for Regulatory Activities, v23.0

^b^Medical Dictionary for Regulatory Activities, v26.0

Corrected version of Table 1

**Table 1:** Summary of TEAEs and ADRs at 12 months [15]^a^ vs those occurring from > 12–36 months^b^ (safety population)

	Phase 2 study [15] (up to 12 months) (*N* = 29)	Extension study (> 12–36 months) (*N* = 27)
At least one ADR	29 (100)	25 (92.6)
At least one grade 1 ADR	20 (69.0)	15 (55.6)
At least one grade 2 ADR	9 (31.0)	8 (29.6)
At least one grade ≥ 3 ADR	0	2 (7.4)
At least one serious TEAE	1 (3.4)	3 (11.1)
At least one TEAE of special interest	9 (31.0)	7 (25.9)
ADR leading to treatment discontinuation	1 (3.4)	0
*ADRs occurring in* ≥ *10% of patients (in either study) by Preferred Term*		
Alopecia	16 (55.2)	1 (3.7)
Fatigue	8 (27.6)	1 (3.7)
Influenza-like illness	8 (27.6)	0
Alanine aminotransferase increased	6 (20.7)	1 (3.7)
Beta-2 microglobulin urine increased	6 (20.7)	7 (25.9)
Aspartate aminotransferase increased	5 (17.2)	1 (3.7)
Diarrhea	5 (17.2)	1 (3.7)
Liver function test abnormal	4 (13.8)	1 (3.7)
Myalgia	4 (13.8)	3 (11.1)
Pyrexia	4 (13.8)	2 (7.4)
Anemia	3 (10.3)	5 (18.5)
Arthralgia	3 (10.3)	0
Gamma glutamyl transferase increased	3 (10.3)	0
White blood cell count decreased	0	7 (25.9)
Injection site reaction	3 (10.3)	0
Malaise	3 (10.3)	3 (11.1)
*TEAEs of special interest by System Organ Class and Preferred Term*		
Endocrine disorders	3 (10.3)	2 (7.4)
Hypothyroidism	2 (6.9)	2 (7.4)
Silent thyroiditis	1 (3.4)	0
Psychiatric disorders	**1 (3.4)**	0
Anxiety	1 (3.4)	0
Eye disorders	1 (3.4)	1 (3.7)
Retinal hemorrhage	1 (3.4)	1 (3.7)

Data are *n* (%)

Number and percentage of TEAEs in the present study represent new events confirmed during the extension study (i.e., from > 12–36 months); events that occurred during the original 12-month study were not included. Patients who experienced an event during the original study that resolved and then experienced another event during the extension study were included

*ADR* adverse drug reaction, *TEAE* treatment-emergent adverse event

^a^Medical Dictionary for Regulatory Activities, v23.0

^b^Medical Dictionary for Regulatory Activities, v26.0

