# Supplementary material for: Long-term safety and efficacy of ropeginterferon alfa-2b in Japanese patients with polycythemia vera
Source: Int J Hematol. 2024 Oct 3;120(6):675–83. doi: 10.1007/s12185-024-03846-5 (PMC11588802; doi:10.1007/s12185-024-03846-5)
Supplement: Supplementary file 1 — Supplementary file1 (PDF 118 kb) [file 12185_2024_3846_MOESM1_ESM.pdf]

Supplementary Figures

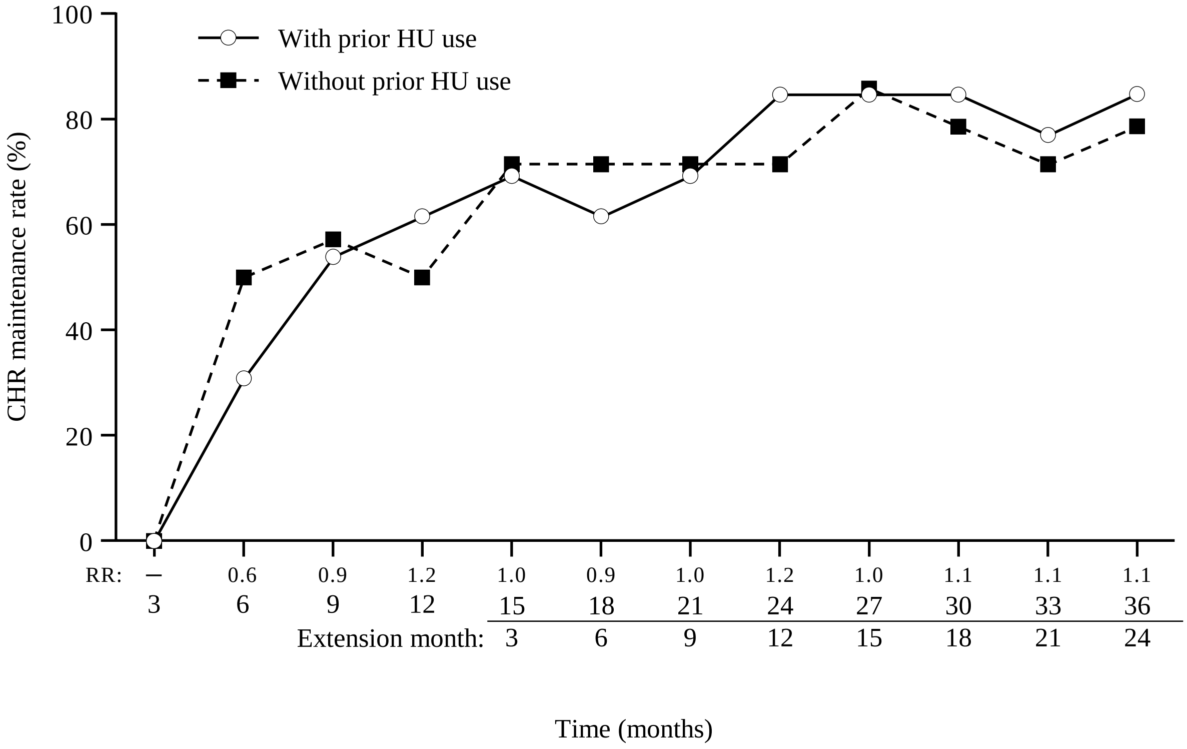

Fig. S1. Maintenance of CHR with/without prior hydroxyurea use (intention-to-treat population, local laboratory data)

CHR, complete hematologic response; HU, hydroxyurea; RR, relative risk

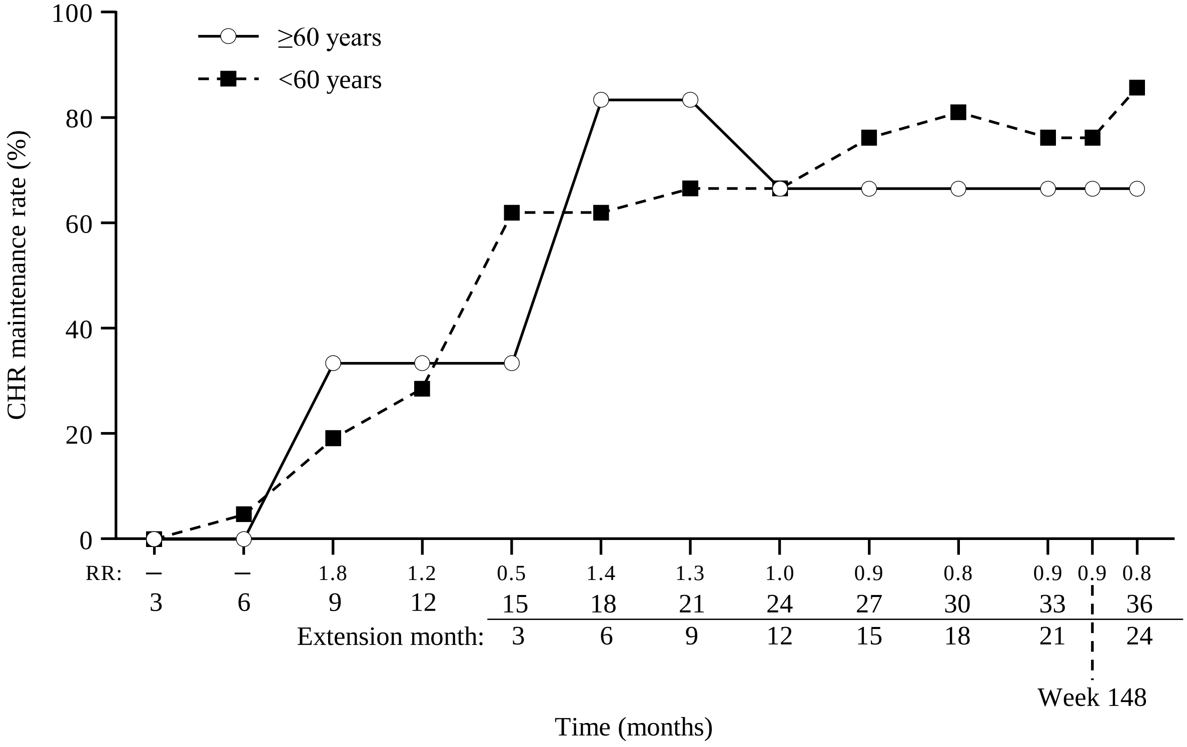

Fig. S2. Maintenance of CHR by age (intention-to-treat population, central laboratory data)

CHR, complete hematologic response; RR, relative risk

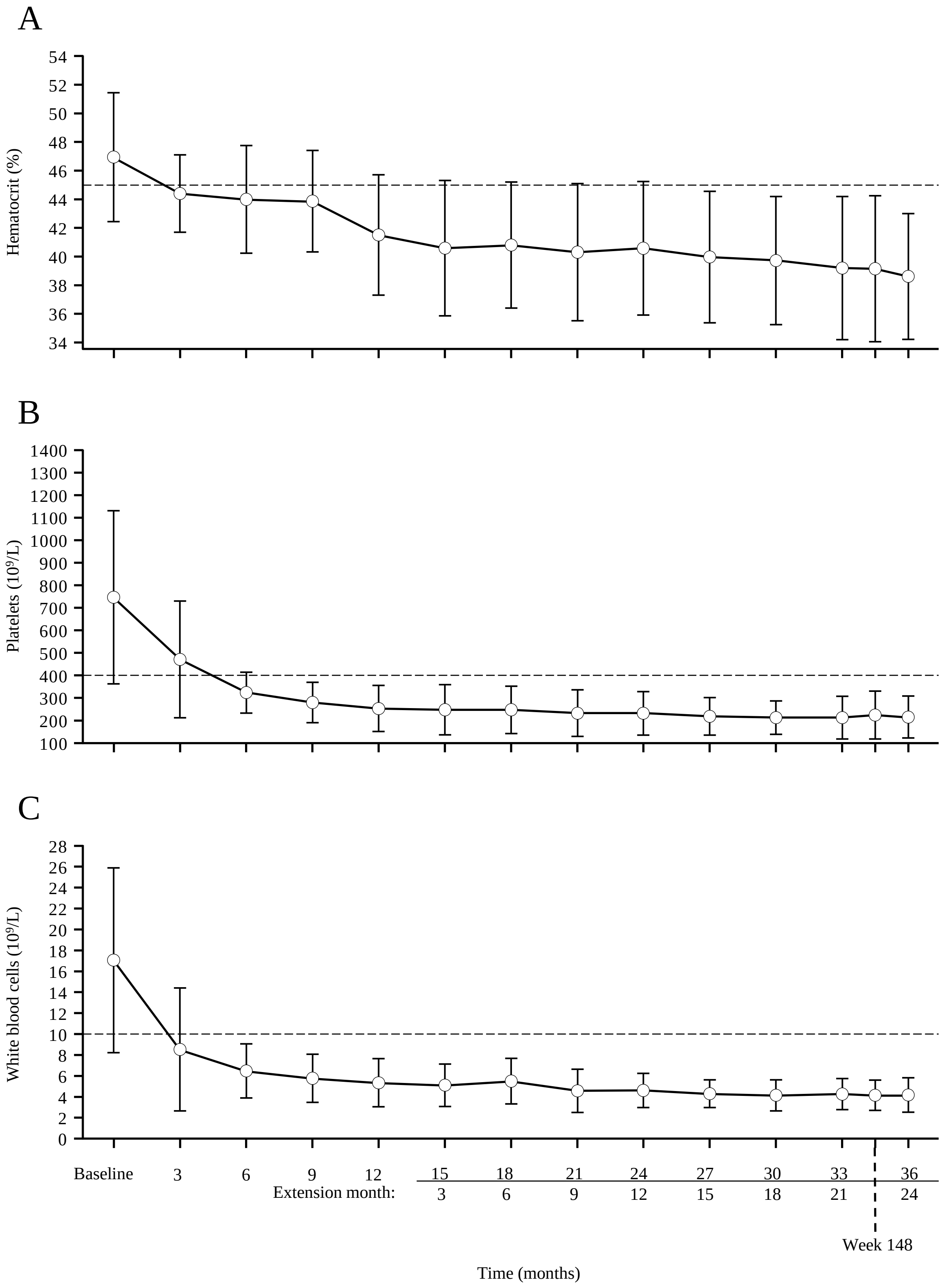

Fig. S3. Monthly mean hematocrit (A), platelets (B), and white blood cells (C) (intention-to-treat population, central laboratory data)
The horizontal dashed line represents the target value. Data are mean ± standard deviation

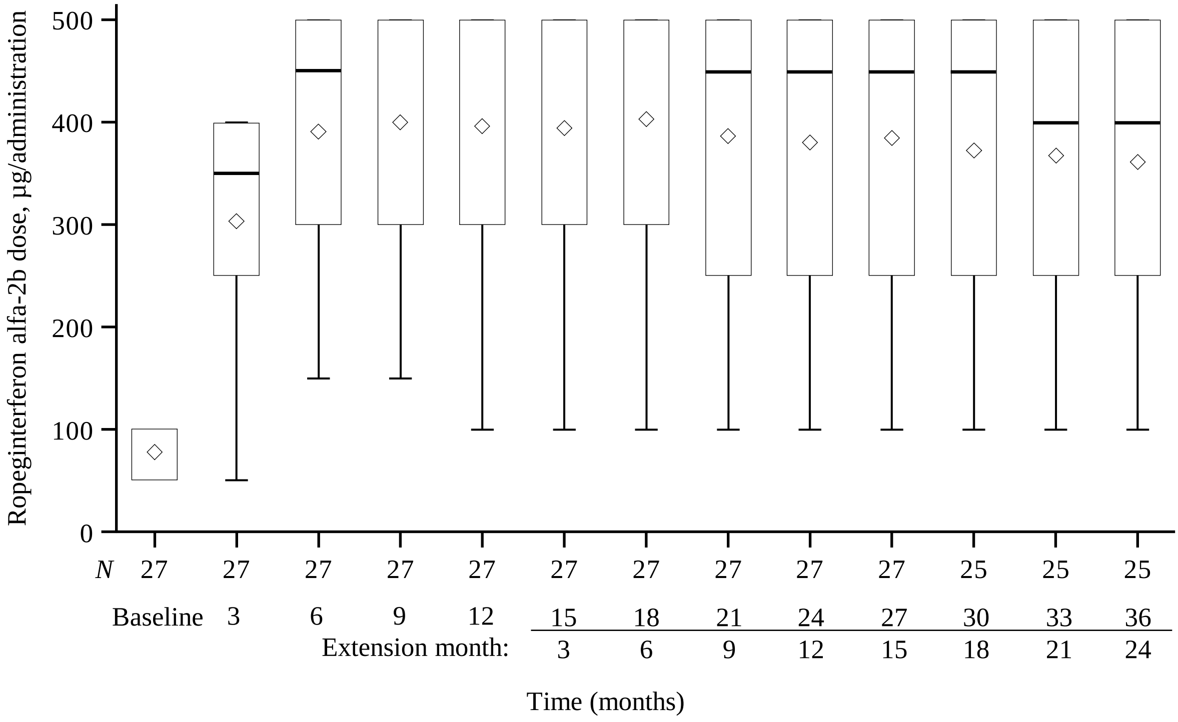

Fig. S4. Mean monthly drug exposure (safety population)

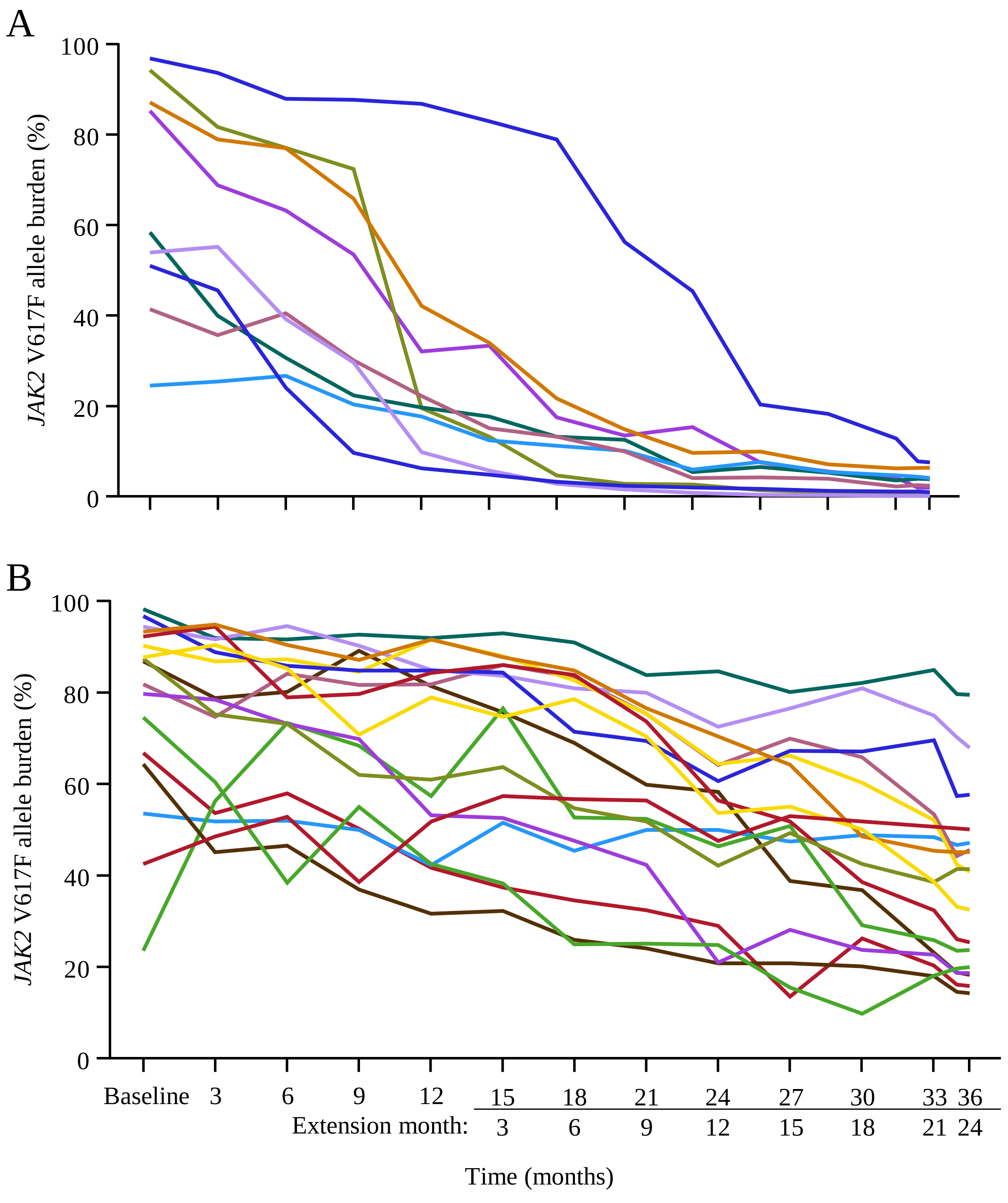

Fig. S5. *JAK2* V617F allele burden for individual patients (A) Data from patients who achieved an allele burden of ≤10% at 36 months, *n* = 9, and (B) patients with an allele burden of >10% at 36 months, *n* = 17. Data from week 148 or week 160 are shown as 36 months.
