# Supplementary material for: Long-term safety and efficacy of ropeginterferon alfa-2b in Japanese patients with polycythemia vera
Source: Int J Hematol. 2024 Oct 3;120(6):675–83. doi: 10.1007/s12185-024-03846-5 (PMC11588802; doi:10.1007/s12185-024-03846-5)
Supplement: Supplementary file 2 — Supplementary file2 (PDF 227 kb) [file 12185_2024_3846_MOESM2_ESM.pdf]

Supplementary Tables

Table S1. Baseline* characteristics of Japanese patients with polycythemia vera (safety population)

Variable	Ropeginterferon alfa-2b (N = 27)
Sex, female	16 (59.3)
Age, years (median [range])	54 (26–72)
Age group, years	
<60	21 (77.8)
≥60	6 (22.2)
Disease duration, years	
<3.1	13 (48.1)
≥3.1	13 (48.1)
Missing	1 (3.7)
ECOG grade	
0	27 (100.0)
1−4	0
Prior hydroxyurea use	
Yes	13 (48.1)
No	14 (51.9)

Data are *n* (%), unless otherwise indicated
ECOG, Eastern Cooperative Oncology Group
* Baseline in original study

Confidential - Not for Public Consumption or Distribution

Table S2. Spleen size (cm^2) and changes from baseline up to 36 months (intention-to-treat population)

		Ropeginterferon alfa-2b (*N* = 27)		
Visit	Statistic	Value	Change from baseline	Percent change from baseline
Baseline	Mean (SD)	64.1 (29.2)		
	Median (range)	51.8 (31.0–143.4)		
Month 3	Mean (SD)	66.2 (30.6)	2.1 (9.6)	4.6 (15.0)
	Median (range)	54.6 (29.8–167.7)	2.0 (−22.2–24.3)	3.9 (−20.3–50.4)
Month 6	Mean (SD)	66.0 (27.7)	1.8 (10.8)	5.9 (18.5)
	Median (range)	58.8 (32.0–151.9)	1.3 (−26.2–18.1)	3.2 (−22.9–53.2)
Month 9	Mean (SD)	61.5 (28.5)	−2.6 (12.1)	−2.2 (17.4)
	Median (range)	54.5 (28.4–151.5)	−1.3 (−36.3–16.1)	−3.1 (−31.8–38.4)
Month 12	Mean (SD)	59.1 (27.7)	−5.0 (15.1)	−5.2 (19.5)
	Median (range)	55.0 (29.8–155.3)	−2.1 (−51.0–20.2)	−4.2 (−44.6–36.2)
Month 18 (Extension month 6)	Mean (SD)	56.2 (27.3)	−7.9 (15.7)	−10.1 (23.1)
	Median (range)	49.4 (20.8–123.1)	−3.1 (−35.2–24.2)	−5.0 (−58.7–29.2)
Month 24 (Extension month 12)	Mean (SD)	52.6 (22.5)	−11.5 (14.8)	−15.2 (16.6)
	Median (range)	48.0 (27.9–111.9)	−6.1 (−58.3–7.7)	−13.1 (−51.1–9.2)
Month 30 (Extension month 18) (*N* = 26)	Mean (SD)	54.0 (22.9)	−11.1 (16.9)	−13.6 (20.2)
	Median (range)	51.3 (26.7–113.8)	−6.1 (−69.0–9.3)	−10.6 (−60.5–20.8)
Month 33 (Extension month 21) (*N* = 1)	Mean (SD)	63.5 (NA)	−19.6 (NA)	−23.6 (NA)
	Median (range)	63.5 (63.5–63.5)	−19.6 (−19.6–−19.6)	−23.6 (−23.6–−23.6)
Month 36 (Extension month 24) (*N* = 25)	Mean (SD)	51.2 (26.3)	−13.2 (18.0)	−18.5 (19.5)
	Median (range)	44.5 (24.9–131.9)	−9.2 (−69.7–20.3)	−15.5 (−61.0–18.2)

NA, not applicable; SD, standard deviation

Confidential - Not for Public Consumption or Distribution

Table S3. Summary of duration of drug exposure (safety population)

Variable	Statistic	Ropeginterferon alfa-2b ($N = 27$)
Duration of drug exposure, days	Mean (SD)	1162.8 (140.0)
	Median (range)	1208.0 (780–1275)
Duration of drug exposure, weeks	Mean (SD)	166.1 (20.0)
	Median (range)	172.6 (111.4–182.1)
Duration of drug exposure, months	Mean (SD)	38.1 (4.6)
	Median (range)	39.6 (25.6–41.8)
Maximum dose level, µg	Mean (SD)	409.3 (120.9)
	Median (range)	500.0 (150–500)
Cumulative dose, µg	Mean (SD)	26,690.7 (11,661.3)
	Median (range)	27,100.0 (9100–44,200)
Mean daily dose, µg/day	Mean (SD)	23.0 (9.6)
	Median (range)	23.7 (7.5–35.5)
Mean dose level, µg/4 weeks	Mean (SD)	644.7 (268.2)
	Median (range)	663.2 (209.2–993.2)

SD, standard deviation

Confidential - Not for Public Consumption or Distribution

Table S4. Summary of treatment-emergent adverse events by prior hydroxyurea treatment (safety population)

	Ropeginterferon alfa-2b ($N = 27$)		
Number of patients with AE[a]	With prior HU use	Without prior HU use	Total
At least one TEAE	13 (100.0)	14 (100.0)	27 (100.0)
Related to study treatment[b]	11 (84.6)	14 (100.0)	25 (92.6)
At least one grade ≥3 toxicity TEAE	5 (38.5)	2 (14.3)	7 (25.9)
Related to study treatment[b]	1 (7.7)	1 (7.1)	2 (7.4)
At least one SAE	3 (23.1)	0	3 (11.1)
Related to study treatment[b]	0	0	0
At least one AESI	1 (7.7)	6 (42.9)	7 (25.9)
Related to study treatment[b]	0	4 (28.6)	4 (14.8)
At least one AE leading to major PV-related cardiovascular event	0	0	0
Related to study treatment[b]	0	0	0
At least one AE leading to psychiatric disorder	0	1 (7.1)	1 (3.7)
Related to study treatment[b]	0	0	0
At least one AE leading to ocular events	0	2 (14.3)	2 (7.4)
Related to study treatment[b]	0	1 (7.1)	1 (3.7)
At least one AE leading to immunologic reaction	0	3 (21.4)	3 (11.1)
Related to study treatment[b]	0	3 (21.4)	3 (11.1)
At least one AE leading to grade ≥2 symptoms[c]	1 (7.7)	0	1 (3.7)
Related to study treatment[b]	0	0	0
Patients with hemorrhagic event	0	0	0
Related to study treatment[b]	0	0	0
AE leading to study treatment discontinuation	1 (7.7)	0	1 (3.7)
Related to study treatment[b]	0	0	0
Patients with SAE leading to study treatment discontinuation	1 (7.7)	0	1 (3.7)
Related to study treatment[b]	0	0	0
Patients with AE with fatal outcome	0	0	0
Related to study treatment[b]	0	0	0

[a]Patients may be counted in more than one category
[b]Includes AEs judged by the investigator to be related to the study drug, and AEs with unknown relationship
[c]Symptoms include palpitations, tachycardia, headache, dizziness, nausea, vomiting, and diarrhea
Data are *n* (%)
AE, adverse event; AESI, AE of special interest; HU, hydroxyurea; PV, polycythemia vera; SAE, serious AE; TEAE, treatment-emergent AE

Confidential - Not for Public Consumption or Distribution

Table S5. Summary of details of dose adjustments/interruptions (safety population)

Parameter	Ropeginterferon alfa-2b (*N* = 27)
Patients without dose change	1 (3.7)
Response achieved	1 (3.7)
Patients with dose reduction	10 (37.0)
Patients with 50-µg reduction	3 (11.1)
Patients with 100-µg reduction	2 (7.4)
Patients with ≥150-µg reduction	5 (18.5)
Reasons leading to dose reduction[a]	
AE suspected to be related to study drug	10 (37.0)
Alanine aminotransferase increased	1 (3.7)
Alopecia	1 (3.7)
Anemia	2 (7.4)
Fatigue	1 (3.7)
Liver function test abnormal	1 (3.7)
Lymphopenia	1 (3.7)
Malaise	1 (3.7)
Platelet count decreased	2 (7.4)
Thrombocytopenia	1 (3.7)
Ventricular hypokinesia	1 (3.7)
White blood cell count decreased	4 (14.8)
Number of patients with dose interruption	5 (18.5)
Patients with one interruption	2 (7.4)
Patients with two interruptions	2 (7.4)
Patients with three or more interruptions	1 (3.7)
Reasons leading to dose interruption[a]	
AE suspected to be related to study drug	5 (18.5)
Alanine aminotransferase increased	1 (3.7)
Enterocolitis	1 (3.7)
Herpes zoster	1 (3.7)
Nasopharyngitis	1 (3.7)
Ventricular hypokinesia	1 (3.7)
White blood cell count decreased	1 (3.7)

[a]Patients may have had more than one reason for dose reduction/interruption
Data are *n* (%)
AEs were coded according to the Medical Dictionary for Regulatory Activities, version 26.0
AE, adverse event

Confidential - Not for Public Consumption or Distribution
